# The Global Parkinson’s Disease Genetics (GP2) Genome Browser

**DOI:** 10.64898/2025.12.29.25343143

**Published:** 2025-12-31

**Authors:** Zih-Hua Fang, Riley H. Grant, Dan Vitale, Carlos F. Hernandez, Samantha Hong, Hampton L. Leonard, Mary B. Makarious, Lara M. Lange, Matthew Solomonson, Peter Heutink, Allison A. Dilliott, Kamalini Ghosh Galvelis, Mike A. Nalls, Andrew B. Singleton, Cornelis Blauwendraat, the Global Parkinson’s Genetics Program (GP2)

**Affiliations:** 1 DataTecnica, Washington, DC, USA; 2 German Center for Neurodegenerative Disease (DZNE), Tübingen, Germany; 3 Program in Medical and Population Genetics, Broad Institute of MIT and Harvard, Cambridge, MA, USA; 4 Universidad del Desarrollo, Centro de Genética y Genómica, Facultad de Medicina Clínica Alemana, Santiago 7610658, Chile; 5 Center for Alzheimer's and Related Dementias (CARD), National Institute on Aging and National Institute of Neurological Disorders and Stroke, National Institutes of Health, Bethesda, MD, USA; 6 Laboratory of Neurogenetics, National Institute on Aging, Bethesda, Maryland, USA; 7 Global Parkinson's Genetics Program, MD, USA; 8 Parkinson’s Foundation, New York, NY, USA; 9 Coalition for Aligning Science, Chevy Chase, MD, USA

**Keywords:** Parkinson’s disease, browser, sequencing, genetics

## Abstract

**Background:**

Large-scale sequencing initiatives have generated extensive genomic resources essential for variant interpretation, yet their effective use often requires bioinformatics expertise. To support identification of Parkinson’s disease (PD) risk and disease-causing variants, we developed an open-access, summary-level genomic data browser.

**Methods:**

We performed uniform joint variant calling to harmonize whole-genome sequencing (WGS) data from AMP-PD Release 4, GP2 Data Releases, and additional controls from the Alzheimer’s Disease Sequencing Project. Clinical exome sequencing (CES) data from GP2 Release 8 was also included.

**Results:**

The integrated dataset includes 31,665 WGS and 9,559 CES samples, spanning eleven ancestries and over 300 million variants.

**Conclusion:**

The GP2 Genome Browser is a lightweight, flexible platform providing intuitive gene- and variant-level summaries with ancestry-stratified allele frequencies and functional annotations. It is open source and freely accessible at https://gp2.broadinstitute.org, enabling broad access to PD genomic data and supporting global research efforts.

## Introduction

Precise molecular mechanisms underlying Parkinson’s Disease (PD) remain largely uncertain. The known risk factors include aging, environmental exposures, and complex genetic factors. Advances in genetic research have identified both rare causative and common risk variants, primarily in individuals of European ancestry^1^. Extending the data to large, ancestrally diverse cohorts will improve our understanding of differences in genetic susceptibility to PD across populations, which is crucial for developing efficient therapies that target patients with diverse ancestries.

Whole-genome sequencing provides a more comprehensive view of the genetic architecture by capturing a broad range of genetic variation, including rare variants that may have a greater phenotypic impact. However, analyzing and interpreting these large-scale data requires substantial bioinformatics expertise, underscoring the importance of continued data aggregation and harmonization to improve variant interpretation^2^. A prior initiative, the Parkinson’s Disease DNA Variant Browser^3^, primarily featured data from individuals of European ancestry. Building on this foundation, we leveraged whole-genome (WGS) and clinical-exome (CES) sequencing data available from the Global Parkinson’s Genetics Program (GP2) Data Releases, Accelerating Medicines Partnership - Parkinson Disease (AMP-PD, https://amp-pd.org/) and additional controls from the Alzheimer’s Disease Sequencing Project^4^. Using these datasets, we developed the GP2 Genome Browser, which integrates and displays variant- and gene-level data across diverse genetic ancestries.

## Methods

### Whole-genome sequencing (WGS) data

We harmonized the WGS data available from AMP-PD release 4, GP2 Data Release 10 (DOI: 10.5281/zenodo.15748014)^5^ and additional 4,278 controls of European ancestry available from the Alzheimer’s Disease Sequencing Project (DOI: 10.60859/z6z9-9692)^4^ by generating single-sample variant calls from the alignment files with DeepVariant v.1.6.1^6^ (https://github.com/google/deepvariant) followed by joint genotyping of single nucleotide variants (SNVs) and short indels using GLnexus v1.4.3 (https://github.com/dnanexus-rnd/GLnexus) with the preset DeepVariant WGS configuration^7^. We set genotypes to be missing after genotype quality control, defined as genotype quality >= 10, read depth >= 5, and heterozygous allele balance between 0.2 and 0.8, and retained high-quality variants with a call rate >= 0.95. After sample quality control using the quality metrics defined by AMP-PD^8^, we retained 31,665 samples for downstream analyses. We used KING v.2.3.0 (https://www.kingrelatedness.com, RRID:SCR_009251)^9^ to infer relatedness up to the second-degree relatives to confirm known relationships and identify cryptic familial relationships. Genetic ancestry was determined using GenoTools v1.2.3 (https://github.com/GP2code/GenoTools) with the default settings^10^. Variant annotation was performed with Ensembl Variant Effect Predictor v111 (http://www.ensembl.org/info/docs/tools/vep/index.html, RRID:SCR_007931)^11^. Intergenic variants were excluded from the browser display. Allele frequencies for the remaining variants were calculated and stratified by phenotype and genetic ancestry. PD patients were defined as individuals diagnosed with PD, excluding those enrolled via targeted recruitment of known mutation carriers (such as *LRRK2* and *GBA1*). Controls were defined as healthy individuals, excluding those recruited as known mutation carriers, individuals recruited through family-based designs, and those with a positive family history of PD. Related individuals were retained because the dataset was generated for association studies using mixed logistic regression models, with the results planned for integration into the browser at a later stage. The remaining individuals were grouped together as ‘Other’ phenotypes for allele frequency calculation. This ‘Other’ phenotypes group contains other primary phenotypes, including but not limited to prodromal stages of PD (e.g., hyposmia and REM sleep behavior disorder), atypical parkinsonism, other neurodegenerative or movement disorders, and dementia; for further details, see the GP2 release notes (https://gp2.org/updates/?post_type=Data%20Release#results).

### Clinical-exome sequencing

We included 10,454 samples with clinical-exome data available from PD GENEration^12^ as part of GP2’s Data Release 8 (DOI 10.5281/zenodo.13755496)^13^. The sequence data processing followed the same pipeline as the WGS data mentioned above. We performed joint-genotyping using GLnexus (v1.4.3) with the preset DeepVariant WES configuration and applied the same criteria for genotype, sample, and variant quality control. After excluding CES samples identified as genetic duplicates of individuals in the WGS dataset, a final set of 9,559 samples was retained for downstream frequency calculation.

## Results

We performed uniform joint variant calling to harmonize WGS data from AMP-PD Release 4, GP2 Data Release 10^5^, and additional controls of European ancestry available from the Alzheimer’s Disease Sequencing Project^4^. Additionally, we included the CES data from GP2 Data Release 8^13^ for calculating allele frequency. After excluding samples that failed quality control or were duplicated, and removing variants that failed quality control or were located in intergenic regions, we retained 303,505,959 variants from 31,665 individuals with WGS data and 1,499,072 variants from 9,559 individuals with CES data. The WGS cohort comprised individuals of European (71.4%), East Asian (8.0%), Ashkenazi Jewish (6.1%), African (5.3%), and other ancestries, while the CES cohort was predominantly European (82.2%) (Table 1).

**Figure 1.**
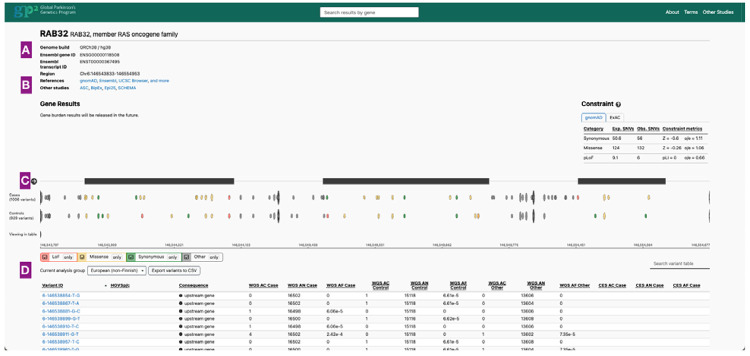
Gene page. (A) Gene information (B) Links to various external resources, as well as constraint information available from gnomAD 4.1 (C) The position of the variants in the selected genetic ancestry stratified by case and control (D) A table of all variants is provided with additional annotation information and links to variant pages.

**Figure 2.**
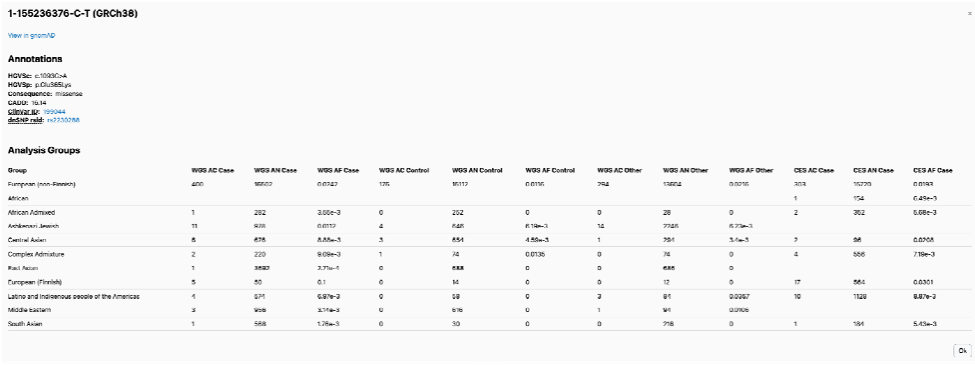
Variant page. A set of variant annotations is displayed, including links to dbSNP and ClinVar. Allele frequency information is displayed for each genetic ancestry and phenotypes.

The browser adopts a gene page interface similar to that of the Genome Aggregation Database (gnomAD, http://gnomad.broadinstitute.org, RRID:SCR_014964)^14^. The front page includes a search bar with autocomplete functionality allowing users to search gene symbols and Ensembl gene IDs. The gene page provides an overview of gene-level information (Figure 1A). It integrates reference data from external resources, including gnomAD and other large-scale case-control studies of neurological phenotypes, such as the Bipolar Exome (BipEx) sequencing project^15^ and the Epi25 Collaborative^16^, among others (Figure 1B). An exon summary plot illustrates the positions of detected variants within the selected ancestry group and the total number of variants stratified by case and control status, with color coding representing their functional annotations (Figure 1C). Variants detected within the selected gene are also displayed on this page, stratified by genetic ancestry (Figure 1D). The variant page presents a set of annotations for each variant, including the CADD Phred score^17^, ClinVar classification^18^, and dbSNP rsID^19^, along with a table summarizing population frequencies across genetic ancestries and datasets (Figure 2).

We examined known pathogenic and risk variants in established PD genes to validate their distribution across genetic ancestries. In the *LRRK2* gene, the p.G2019S variant—one of the most common pathogenic variants—was detected across several ancestries but was absent in African admixed, Central Asian, East Asian, South Asian, and Finnish European populations. The highest allele frequency among PD patients (0.04) was observed in individuals of Ashkenazi Jewish ancestry, followed by Middle Eastern ancestry (0.01) in the WGS dataset. These findings are consistent with previous reports showing that *LRRK2* p.G2019S is particularly common in Ashkenazi Jewish and North African Berber Arab populations^20,21^. Conversely, the East Asian–specific risk variant p.G2385R in *LRRK2* was identified in non-Finnish European, Central Asian, and East Asian populations, with a markedly higher allele frequency in East Asian PD patients (0.05) compared to non-Finnish European PD patients (1.21×10 ) in the WGS dataset, also consistent with prior findings^22^. Additionally, the recently identified *RAB32* p.S71R variant^23,24^ was detected in non-Finnish European and Middle Eastern populations but was absent in all other ancestries. Taken together, these findings demonstrate that the browser can facilitate the investigation of both shared and ancestry-specific patterns in the prevalence and spectrum of SNVs and indels relevant to PD research.

## Discussion

We present the GP2 Genome Browser (https://gp2.broadinstitute.org), a public platform that allows for rapid querying of specific genes and variants within the largest and most diverse sequencing collection assembled for PD research (13,063 PD vs 9,912 Others vs 8,690 Control). Upon input of a gene HGNC symbol or Ensembl gene ID, the browser presents the variants found within that gene, categorized by functional consequence with the frequencies stratified across genetic ancestries and phenotypes. For a specific variant, the browser displays several annotations, including functional consequence, allele frequency across genetic ancestries and phenotypes, ClinVar information and dbSNP rsID. These functionalities enable users to retrieve the frequency of a variant of interest (identified in a PD patient or within a family) and compare shared versus ancestry-specific patterns across populations.

The current version of the GP2 Genome Browser has several limitations. First, it currently displays data only from the variants located within upstream and downstream 5Kb of the gene body. While these regions are often the most relevant for identifying disease-causing variants, many non-coding regions also play critical roles in human disease and remain active areas of research^25^. Second, the browser does not currently display zygosity distributions, which are relevant for investigating variants that follow a recessive mode of inheritance. Third, the frequencies for pseudogene-related variants in *GBA1*, including p.A495P, p.L483P, p.D448H, c.1263del, RecNciI, RecTL, and c.1263del+RecTL, might be underestimated when using the standard variant calling pipeline compared to the *GBA1* target caller, Gauchian^26^. Furthermore, some *GBA1* variants may be missed due to false negative calls. Finally, allele frequency estimates may be inflated due to the presence of related individuals in the datasets. Note that the browser is intended to display results from association analyses that use mixed logistic regression models, along with the corresponding datasets used for those analyses.

In summary, we present an online resource designed for the PD research community to efficiently access annotated genomic information on genes and variants through a user-friendly interface, similar to gnomAD—not requiring coding or data science expertise required. Researchers can use the browser to explore variant frequencies between PD patients, PD-related phenotypes, and controls, across genetic ancestries. The browser’s data can also be used to complement other researchers’ own analyses with data from large-scale genomic datasets. We plan to update the browser biannually with future GP2 releases, refine its features, and incorporate association results for both single variants and gene-based burden analyses as they become available.

## Figures and Tables

**Table 1.** Number of participants by genetically determined ancestry included in the whole-genome sequencing (WGS) and clinical exome sequencing (CES) datasets.

	WGS			CES
Ancestry	Case	Control	Other	Case
African Admixed	141	126	14	176
African	809	837	19	77
Ashkenazi Jewish	491	323	1123	0
Complex Admixture	110	37	37	278
Central Asian	338	327	147	48
East Asian	1848	344	343	130
Finnish European	25	7	6	282
Latino and Indigenous people of the Americas	287	29	42	564
Middle Eastern	478	308	47	52
Non-Finnish European	8252	7559	6804	7860
South Asian	284	15	108	92
Total	13063	9912	8690	9559

* Cases were defined as individuals diagnosed with PD, excluding those recruited based on genetic status. Controls were defined as healthy individuals, excluding those recruited based on genetic status or through family-based recruitment. The ‘Other’ phenotypes group contains other primary phenotypes, including but not limited to prodromal stages of PD (e.g., hyposmia and REM sleep behavior disorder), atypical parkinsonism, other neurodegenerative or movement disorders, and dementia; for further details, see the GP2 release notes (https://gp2.org/updates/?post_type=Data%20Release#results).

## References

[1] Schumacher-Schuh A. F. Underrepresented populations in Parkinson’s genetics research: Current landscape and future directions. Mov. Disord. 37, 1593–1604 (2022). 35867623 10.1002/mds.29126 PMC10360137

[2] Naslavsky M. S. Biased pathogenic assertions of loss of function variants challenge molecular diagnosis of admixed individuals. Am. J. Med. Genet. C Semin. Med. Genet. 187, 357–363 (2021). 34189818 10.1002/ajmg.c.31931

[3] Kim J. J. The Parkinson’s disease DNA variant browser. Mov. Disord. 36, 1250–1258 (2021). 33497488 10.1002/mds.28488 PMC8248407

[4] Leung Y. Y. Alzheimer’s Disease Sequencing Project release 4 whole genome sequencing dataset. Alzheimers. Dement. 21, e70237 (2025). 40407102 10.1002/alz.70237 PMC12100500

[5] Leonard H. Global Parkinson’s Genetics Program data release 10. Zenodo 10.5281/ZENODO.15748014 (2025).

[6] Poplin R. A universal SNP and small-indel variant caller using deep neural networks. Nat. Biotechnol. 36, 983–987 (2018). 30247488 10.1038/nbt.4235

[7] Yun T. Accurate, scalable cohort variant calls using DeepVariant and GLnexus. Bioinformatics 36, 5582–5589 (2021). 33399819 10.1093/bioinformatics/btaa1081 PMC8023681

[8] Iwaki H. Accelerating Medicines Partnership: Parkinson’s Disease. Genetic Resource. Mov. Disord. 36, 1795–1804 (2021). 33960523 10.1002/mds.28549 PMC8453903

[9] Manichaikul A. Robust relationship inference in genome-wide association studies. Bioinformatics 26, 2867–2873 (2010). 20926424 10.1093/bioinformatics/btq559 PMC3025716

[10] Vitale D. GenoTools: An Open-Source Python Package for Efficient Genotype Data Quality Control and Analysis. bioRxiv (2024) doi:10.1101/2024.03.26.586362. PMC11708233 39566101

[11] McLaren W. The Ensembl Variant Effect Predictor. Genome Biol. 17:122, (2016). 27268795 10.1186/s13059-016-0974-4 PMC4893825

[12] Cook L. Parkinson’s disease variant detection and disclosure: PD GENEration, a North American study. Brain 147, 2668–2679 (2024). 39074992 10.1093/brain/awae142 PMC11292896

[13] Leonard H. Global Parkinson’s Genetics Program data release 7. Zenodo 10.5281/ZENODO.10962119 (2024).

[14] Chen S. A genomic mutational constraint map using variation in 76,156 human genomes. Nature 625, 92–100 (2024). 38057664 10.1038/s41586-023-06045-0 PMC11629659

[15] Liao C., Talkowski M. & Neale B. 2. Bipex 2.0: Large-scale exome sequencing of over 75,000 individuals identifies novel genetic insights into bipolar disorder. Eur. Neuropsychopharmacol. 75, S56–S57 (2023).

[16] Epi25 Collaborative. Exome sequencing of 20,979 individuals with epilepsy reveals shared and distinct ultra-rare genetic risk across disorder subtypes. Nat. Neurosci. 27, 1864–1879 (2024). 39363051 10.1038/s41593-024-01747-8 PMC11646479

[17] Rentzsch P., Schubach M., Shendure J. & Kircher M. CADD-Splice-improving genome-wide variant effect prediction using deep learning-derived splice scores. Genome Med. 13, 31 (2021). 33618777 10.1186/s13073-021-00835-9 PMC7901104

[18] Landrum M. J. ClinVar: public archive of relationships among sequence variation and human phenotype. Nucleic Acids Res. 42, D980–5 (2014). 24234437 10.1093/nar/gkt1113 PMC3965032

[19] Sherry S. T. dbSNP: the NCBI database of genetic variation. Nucleic Acids Res. 29, 308–311 (2001). 11125122 10.1093/nar/29.1.308 PMC29783

[20] Correia Guedes L. Worldwide frequency of G2019S LRRK2 mutation in Parkinson’s disease: a systematic review. Parkinsonism Relat. Disord. 16, 237–242 (2010). 19945904 10.1016/j.parkreldis.2009.11.004

[21] Kmiecik M. J. Genetic analysis and natural history of Parkinson’s disease due to the LRRK2 G2019S variant. Brain 147, 1996–2008 (2024). 38804604 10.1093/brain/awae073 PMC11146432

[22] Shu L., Zhang Y., Sun Q., Pan H. & Tang B. A comprehensive analysis of population differences in LRRK2 variant distribution in Parkinson’s disease. Front. Aging Neurosci. 11, 13 (2019). 30760999 10.3389/fnagi.2019.00013 PMC6363667

[23] Hop P. J. Systematic rare variant analyses identify RAB32 as a susceptibility gene for familial Parkinson’s disease. Nat. Genet. 56, 1371–1376 (2024). 38858457 10.1038/s41588-024-01787-7 PMC11250361

[24] Gustavsson E. K. RAB32 Ser71Arg in autosomal dominant Parkinson’s disease: linkage, association, and functional analyses. Lancet Neurol. 23, 603–614 (2024). 38614108 10.1016/S1474-4422(24)00121-2 PMC11096864

[25] Ellingford J. M. Recommendations for clinical interpretation of variants found in non-coding regions of the genome. Genome Med. 14, 73 (2022). 35850704 10.1186/s13073-022-01073-3 PMC9295495

[26] Toffoli M. Comprehensive short and long read sequencing analysis for the Gaucher and Parkinson’s disease-associated GBA gene. Commun Biol 5, 670 (2022). 35794204 10.1038/s42003-022-03610-7 PMC9259685

